# Hierarchically Developed Ni(OH)_2_@MgCo_2_O_4_ Nanosheet Composites for Boosting Supercapacitor Performance

**DOI:** 10.3390/nano13081414

**Published:** 2023-04-19

**Authors:** Hammad Mueen Arbi, Ganesh Koyyada, Yedluri Anil Kumar, Dasha Kumar Kulurumotlakatla, Jae Hong Kim, Md Moniruzzaman, Salem Alzahmi, Ihab M. Obaidat

**Affiliations:** 1 Department of Physics, United Arab Emirates University, Al Ain P.O. Box 15551, United Arab Emirates; 2 Department of Chemical Engineering, Yeungnam University, 214-1, Daehak-ro 280, Gyeongsan 712-749, Republic of Korea; 3 Department of Chemical & Petroleum Engineering, United Arab Emirates University, Al Ain P.O. Box 15551, United Arab Emirates; 4 National Water and Energy Center, United Arab Emirates University, Al Ain P.O. Box 15551, United Arab Emirates; 5 Graduate School of Convergence Science, Pusan Nationfivel University, San 30 Jangjeon-dong, Geumjeong-gu, Busan 609-735, Republic of Korea; 6 Department of Chemical and Biological Engineering, Gachon University, 1342 Seongnam-daero, Seongnam-si 13120, Republic of Korea

**Keywords:** hybrid structure, Ni(OH)_2_@MgCo_2_O_4_ composites, electrode, supercapacitors, battery-type, high performance

## Abstract

MgCo_2_O_4_ nanomaterial is thought to be a promising candidate for renewable energy storage and conversions. Nevertheless, the poor stability performances and small specific areas of transition-metal oxides remain a challenge for supercapacitor (SC) device applications. In this study, sheet-like Ni(OH)_2_@MgCo_2_O_4_ composites were hierarchically developed on nickel foam (NF) using the facile hydrothermal process with calcination technology, under carbonization reactions. The combination of the carbon–amorphous layer and porous Ni(OH)_2_ nanoparticles was anticipated to enhance the stability performances and energy kinetics. The Ni(OH)_2_@MgCo_2_O_4_ nanosheet composite achieved a superior specific capacitance of 1287 F g^−1^ at a current value of 1 A g^−1^, which is higher than that of pure Ni(OH)_2_ nanoparticles and MgCo_2_O_4_ nanoflake samples. At a current density of 5 A g^−1^, the Ni(OH)_2_@MgCo_2_O_4_ nanosheet composite delivered an outstanding cycling stability of 85.6%, which it retained over 3500 long cycles with an excellent rate of capacity of 74.5% at 20 A g^−1^. These outcomes indicate that such a Ni(OH)_2_@MgCo_2_O_4_ nanosheet composite is a good contender as a novel battery-type electrode material for high-performance SCs.

## 1. Introduction

With the increasing demand for future-generation renewable energy storage cells, supercapacitors (SCs) are holding on as one of the most favored electrochemical devices, due to their ability to provide greater charge/discharge rates, prolonged cycling life, and superior power densities [1,2,3]. Nevertheless, most SCs are limited by lower energy densities that significantly hamper their further application. As established by the equation (E = 1/2CV^2^), work voltage and electrode samples have pivotal roles in determining the energy density [4]. In order to attain greater energy densities, a hybrid SC setup could be designed to increased work voltages [5,6]. SCs possess a battery-type faradaic sample as the energy key and a capacitor nanomaterial as the power initiator [7]. Moreover, constructing unique electrodes to achieve greater specific capacities has been contemplated as a way to enhance energy densities [8,9]. Thus, there is a strong desire to develop unique electrode samples with superior electrochemical activities.

Transition-metal oxides, metal hydroxides, and polymer-based conductors have been extensively utilized as superior electrodes owing to their greater specific theoretical capacities [10,11]. Binary-transition-metal oxides (BTMOs) have gained significant attention as unique sample materials because of their varied oxidation states, cheap prices, and simple preparations, in addition to being eco-friendly. Cobalt-type spinel BTMOs that include CoMnO_4_ [12], MnCo_2_O_4_ [13], CuCo_2_O_4_ [14], and MgCo_2_O_4_ [15] are being investigated as possible SCs. Because of their excellent theoretical capacitance and sufficient reserves of innate magnesium MgCo_2_O_4_ composites have been considered for SCs and Li-ion batteries [16]. Nevertheless, in practical devices, MgCo_2_O_4_ electrodes alone hardly exhibit a superior theoretical capacity (~3120 F/g) due to their simple nanostructure, small surface region, and weak conductivities [17,18]. To enhance the performance limit of MgCo_2_O_4_ electrodes, it is imperative that a rational and unique design be constructed to improve the energy storage properties of MgCo_2_O_4_-type SCs [19,20].

On other hand, nickel hydroxide Ni(OH)_2_ has been the object of much consideration because of its superior theoretical capacities, superior electrochemical activities, and cheap price [21]. The unique Ni(OH)_2_ nanoparticle that develops on the electrode interface would extend the specific area with the electrolytes and condense the ion diffusion routes to optimize its energy-storing performance [22,23,24]. Meng et al. prepared a ZnCo_2_S_4_/Ni(OH)_2_ sample with a great capacitance of 2156 F g^−1^ at 1 A g^−1^ and solid stability performances (94.3% retained over 3000 cycles) [25]. Liu et al. prepared MnCo-LDHs@Ni(OH)_2_ via an easier two-step hydrothermal route that provided a specific capacitance of 2320 F g^−1^ at 3 A g^−1^ [26]. Therefore, the design of Ni(OH)_2_ sheets and MgCo_2_O_4_ nanoflakes is anticipated to effectively enhance the energy storage activities and impart significant capacities. Meanwhile, it has been shown that electrode materials covered by an amorphous carbon layer display improved stability performances and increased structural stabilities [27,28,29].

To explore extremely effective, unique, and reliable electrode samples, both Ni(OH)_2_ particles and MgCo_2_O_4_ nanoflakes were combined to form a Ni(OH)_2_@MgCo_2_O_4_ nanosheet composite structure. In this study, the novel structure of the Ni(OH)_2_@MgCo_2_O_4_ nanosheet was engineered and developed via a hydrothermal process. This recently discovered Ni(OH)_2_@MgCo_2_O_4_ nanosheet composite displayed a specific capacitance of 1287 F g^−1^ at a current density of 1 A g^−1^, which is superior to that of the MgCo_2_O_4_ nanoflakes and Ni(OH)_2_ particles alone. Additionally, the Ni(OH)_2_@MgCo_2_O_4_ nanosheet composite showed a notable cyclic stability of 85.6% over 3500 long cycles. These results demonstrate that the Ni(OH)_2_@MgCo_2_O_4_ nanosheet composite is a potential electrode material candidate for high-performance SCs.

## 2. Experimental Section

### 2.1. Synthesis of MgCo_2_O_4_ Nanoflakes Grown on Ni Foam

MgCo_2_O_4_ nanoflakes were prepared using a hydrothermal method. NF (2 × 4 cm^−2^) pieces were handled with ultrasonication to detach oxide impurities on their interface. Clear solutions were optimized through fully dissolving 0.964 g of Co(NO_3_)_2_⋅6H_2_O, 0.494 g of Mg(NO_3_)_2_⋅6H_2_O, 0.45 g of CO(NH_2_)_2_, 0.083 g of NH_4_F, in 60 mL of distilled water (DI water). After that, the mixed solution and the reacted NF were moved to a well-sealed 150 mL autoclave and held in an oven at 140 °C for 8 h. Then, the autoclave was cooled down to a normal temperature, and the NF pieces were removed from the precursors, washed 3 times with DI water and ethanol, and then heated at 60 °C for 12 h, accompanied by annealing at 350 °C for 2 h at a rate of 6 °C min^−1^. Eventually, the products were acquired and denoted as MgCo_2_O_4_ nanoflakes.

### 2.2. Preparation of Ni(OH)_2_@MgCo_2_O_4_ Nanosheet Composite

Following a typical procedure, 2 mmol NiCl_2_·6H_2_O and 4 mmol urea were put into 20 mL of DI water and stirred for 30 min to obtain a clear solution. The mixed precursor with pieces of MgCo_2_O_4_ nanoflakes loaded on Ni foam was then moved to a 40 mL autoclave and maintained at 110 °C for 3 h. After being cooled down to a normal temperature, the samples were rinsed with ethanol and DI water several times and dried at 70 °C for 5 h in a vacuum. The mass loading of the Ni(OH)_2_@MgCo_2_O_4_ nanosheet composites was 3.7 mg cm^−2^. For comparison, Ni(OH)_2_ was fabricated on Ni foams using a similar procedure. The mass loading of the MgCo_2_O_4_ nanoflake electrode and Ni(OH)_2_ electrode was 3.7 mg cm^−2^ and 1.9 mg cm^−2^, respectively.

### 2.3. Measurements and Characterizations

X-ray powder diffraction (XRD, Bruker D8 Advance with Cu Kα radiation) was used to characterize the crystalline phases of the samples. Field emission scanning electron microscopy (FE-SEM, JSM-7800F) was used to study the morphologies. High-resolution transmission electron microscopy (HRTEM, JEM-2100F at 200 kV) was used to study the microstructures and the elemental compositions. Photoelectron X-ray spectroscopy (XPS; ESCCALAB 250Xi, Busan, Republic of Korea) was used to study the chemical valence states and compositions of samples.

### 2.4. Measurements and Characterizations

All electrochemical measurements were conducted using a 3-electrode configuration in a 3 M KOH aqueous electrolyte. The fabricated samples, a Ag/AgCl electrode, and a platinum wire were utilized as working electrodes, reference electrodes, and counter electrodes, respectively. Galvanostatic charge–discharge (GCD) and cyclic voltammetry (CV) were acquired on a Bio-Logic- SP-150C electrochemical workstation. Electrochemical impedance spectroscopy (EIS) was employed in the frequency width from 0.01 to 100 kHz. The specific capacitance (*C_s_*, F g^−1^) was calculated from the GCD plots using the following equation [30]:

(1) $$C_{S}=\frac{I\times ∆t}{m\times ∆V}$$

where *I* (A), Δt (s), and *m* (g) have their conventional meanings.

## 3. Results and Discussion

**Figure 1:**
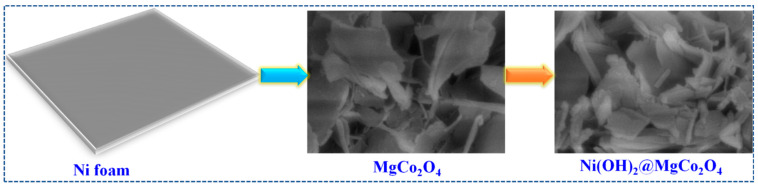
Schematic illustration of the synthesis preparation of Ni(OH)_2_@MgCo_2_O_4_ nanosheet composite grown on Ni foam.

The synthesis procedure of Ni(OH)_2_@MgCo_2_O_4_ is demonstrated in Figure 1. Initially, MgCo_2_O_4_ nanoflakes were developed on Ni foam under simple hydrothermal and annealing technologies. After that, MgCo_2_O_4_ nanoflakes were directly engaged as a skeleton to construct Ni(OH)_2_@MgCo_2_O_4_ through secondary hydrothermal procedures. As an outcome, MgCo_2_O_4_ nanoflakes gradually dissolved and generated fresh Ni(OH)_2_, manifesting a uniform sheet-like Ni(OH)_2_@MgCo_2_O_4_ composite.

**Figure 2:**
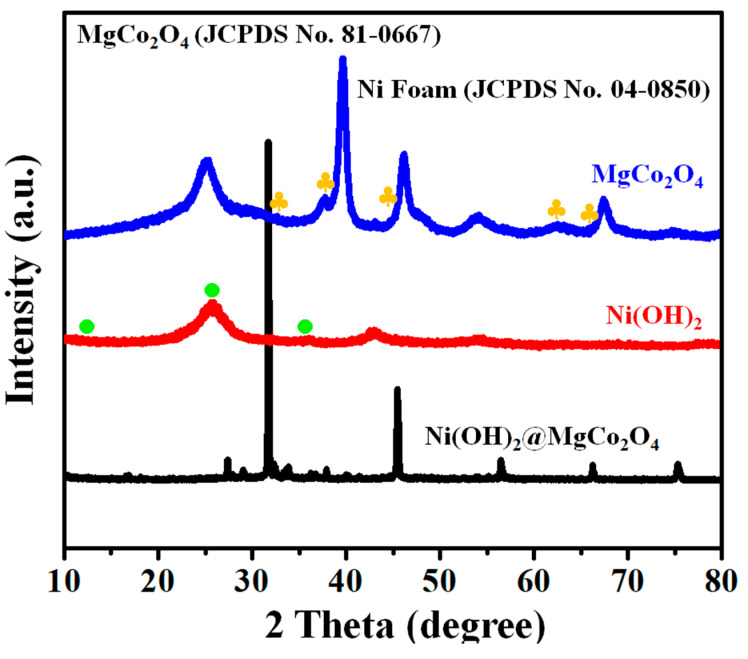
XRD patterns of Ni(OH)_2_ nanoparticle electrode, MgCo_2_O_4_ nanoflake electrode, and Ni(OH)_2_@MgCo_2_O_4_ nanosheet composites grown on Ni foam.

The XRD patterns of the Ni(OH)_2_, MgCo_2_O_4_, and Ni(OH)_2_@MgCo_2_O_4_ nanosheet electrodes are displayed in Figure 2. In the XRD tests of MgCo_2_O_4_, except for the diffraction angles from the nickel foam matrixes that remained, long peaks could be clearly identified in the MgCo_2_O_4_ phases [14]. The diffraction peaks positioned at 31°, 36.8°, 44.6°, 58.89°, and 64.98° were attributed to the (2 2 0), (3 1 1), (4 0 0), (4 2 2), and (4 4 0) lattice planes of MgCo_2_O_4_ (JCPDS NO. 02–1073) [12,16,29]. Additionally, three longer peaks were identified at 2θ = 44.5°, 51.85°, and 76.37° that came from the Ni foam skeleton (JCPDS NO. 04–0850). Meanwhile, the line angles at 11.4°, 23.7°, and 33.4° were matched to Ni(OH)_2_ (JCPDS No. 38-0715) [31,32]. In general, the diffraction angles of the Ni(OH)_2_@MgCo_2_O_4_ nanosheet composite were in good agreement with Ni(OH)_2_ and MgCo_2_O_4_ [28].

**Figure 3:**
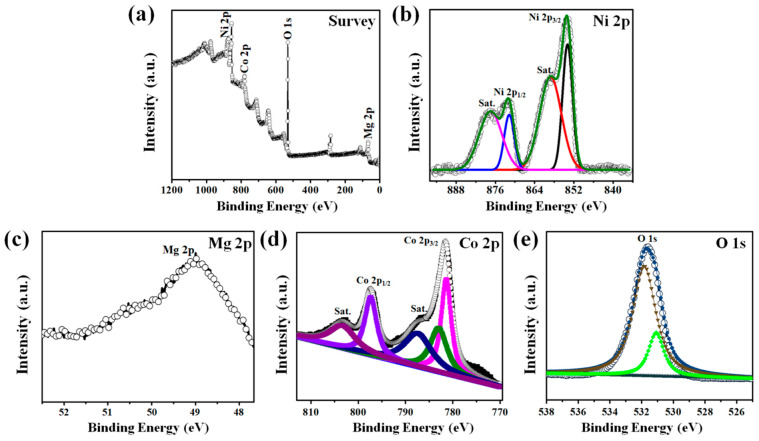
(**a**) XPS full spectra of Ni(OH)_2_@MgCo_2_O_4_ nanosheet composite, and high-resolution XPS spectra of Ni(OH)_2_@MgCo_2_O_4_ nanosheet composite for (**b**) Ni 2p, (**c**) Mg 2p, (**d**) Co 2p, and (**e**) O 1 s.

XPS was utilized to detect the chemical valence states and compositions of each element in the developed Ni(OH)_2_@MgCo_2_O_4_ nanosheet composites. Figure 3a depicts the survey spectra, which illustrate the coexistence of Ni, Mg, Co, and O in the composite sample. The Ni 2p XPS spectra are shown in Figure 3b. The angles situated at 873.5 eV in Ni 2p_1/2_ and 855.6 eV in Ni 2p_3/2_ were ascribed to the Ni^2+^ states, and those located at 876.6 eV in Ni 2p_1/2_ and 858.76 eV in Ni 2p_3/2_ were attributed to the Ni^3+^ states [33,34,35]. The straight spectra of Mg 2p seen in the XPS results are shown in Figure 3c, where the peaks in the binding source at 51.2 eV confirm the existence of magnesium oxides. The Co 2p XPS spectra (Figure 3d) possess two spin orbits and two shake-up satellites (denoted as “Sat.”). The phases at 794.2 eV and 779.4 eV were attributed to Co^3+^, whereas the remaining peaks at 798.4 eV and 782.5 eV were ascribed to Co^2+^ [34]. The percentage of oxidation of cobalt ions was calculated based on the fitted peak areas of all the individual peaks in the Co 2p XPS spectra for the Ni(OH)_2_@MgCo_2_O_4_ nanosheet composites, and was determined to be 41:59 (Co^2+^:Co^3+^). As depicted in Figure 3e, the O 1 s XPS spectra are composed of three different peaks labeled as O1, O2, and O3 [36,37]. The O1 peak at 531.2 eV was assigned to metal-oxidized bonds. The O2 peak at 532.8 eV was attributed to the OH^−^ groups procured from Ni(OH)_2_. The O3 peak at 535.2 eV was ascribed to the chemisorbed oxidized atoms on the interface. In addition, Figure S1 of XPS full spectra of MgCo_2_O_4_ nanoflakes grown on Ni foam.

**Figure 4:**
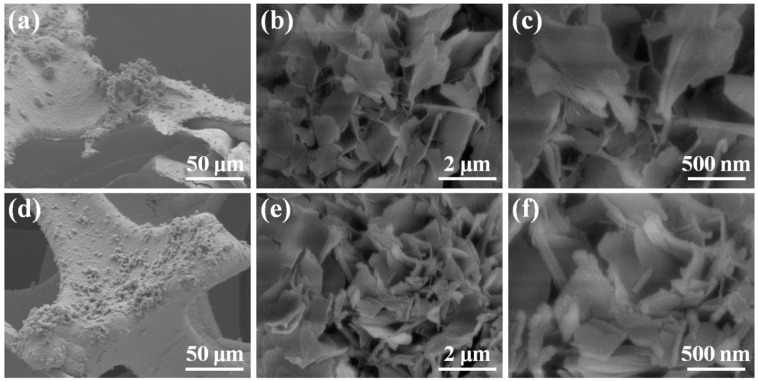
SEM images of the prepared MgCo_2_O_4_ nanoflakes (**a**–**c**) and Ni(OH)_2_@MgCo_2_O_4_ nanosheet composite (**d**–**f**).

The intrinsic nanostructures and morphologies of the Ni(OH)_2_@MgCo_2_O_4_ nanosheet composite were analyzed via FE-SEM. The morphological structure of the MgCo_2_O_4_ nanoflake electrode is shown in Figure 4a–c from lower to higher magnifications. Uniform nanoflakes were grown at the edge of the surface, as shown in Figure 4a. Figure 4b,c indicate that the nanoflakes nearly uniformly covered the initial micro-morphology, and their formation was followed by an annealing process. Figure 4d demonstrates that the nanosheets were grown perpendicular to the skeleton. As illustrated in Figure 4e,f, it is obvious that the interface of the MgCo_2_O_4_ nanoflakes was covered with Ni(OH)_2_ nanoparticles. The buildup of Ni(OH)_2_ nanoparticles did not demolish the intrinsically ordered nanostructure. The nanoflakes and nanosheets would supply efficient transfer channels for the electrolytes during the charge storage procedures. The MgCo_2_O_4_ nanoflakes were fully positioned on Ni(OH)_2_ nanoflakes, which favored the effective conductive, preservative furnishing of MgCo_2_O_4_ nanoflakes with ion transportations in the electrolyte, and also provided safe structural stabilities. In addition, the Ni(OH)_2_@MgCo_2_O_4_ nanosheet composite supplied abundant active sites for the faradic redox process.

**Figure 5:**
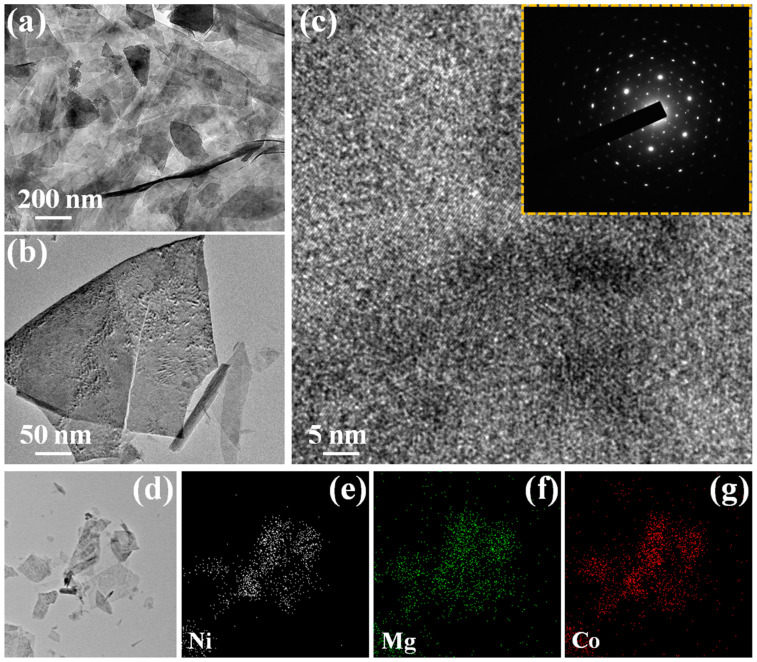
Low-magnification and high-magnification TEM images. (**a**) TEM images of MgCo_2_O_4_ nanoflake electrode; (**b**) TEM images of Ni(OH)_2_@MgCo_2_O_4_ nanosheet composite; (**c**) the HRTEM images of the Ni(OH)_2_@MgCo_2_O_4_ nanosheet composite (the insets of the selected area show the electron diffraction (SAED) patterns); (**d**–**g**) EDS element mapping images of Ni(OH)_2_@MgCo_2_O_4_ nanosheet composite.

The specific microstructures and morphology of the MgCo_2_O_4_ nanoflakes (Figure 5a) and Ni(OH)_2_@MgCo_2_O_4_ nanosheet composite (Figure 5b,c) were obtained using TEM and HRTEM. Figure 5a shows that the MgCo_2_O_4_ nanoflakes existed as the array type, and the nanoflake could be clearly seen. Figure 5b shows the TEM images of a Ni(OH)_2_@MgCo_2_O_4_ nanosheet composite, demonstrating MgCo_2_O_4_ has a sheet-type construction with a diameter range of 50 nm [34]. The HRTEM image of the Ni(OH)_2_@MgCo_2_O_4_ nanosheet composite illustrates well-defined lattice spacings with interplanar fringes of 0.288 nm and 0.244 nm, which were assigned to the (2 2 0) and (3 1 1) planes of MgCo_2_O_4_ [18,29]. The SAED image (inset in Figure 5c) obtained from the nanosheets reveals their polycrystalline characteristic, which greatly enhances the active sites needed for the Faraday capacity process. As shown in Figure 5d–g, the elements of Ni, Mg, and Co were homogeneously coated in the whole nanosheet, indicating the uniform coexistence of a Ni(OH)_2_@MgCo_2_O_4_ nanosheet composite.

### Electrochemical Properties of Electrode Materials

**Figure 6:**
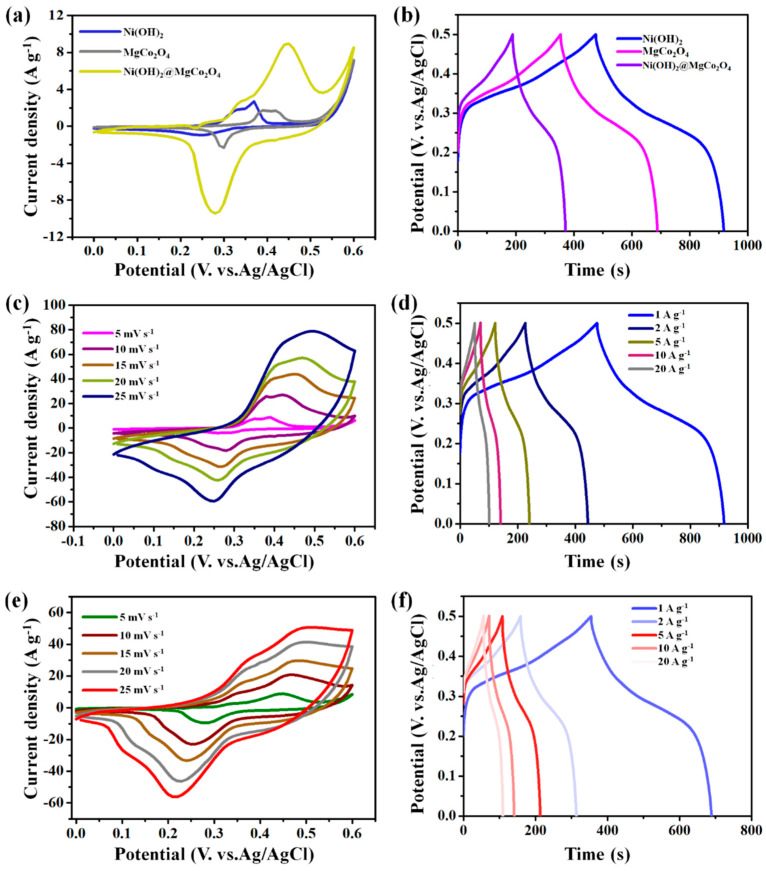
(**a**) Comparison of the CV plots of Ni(OH)_2_ nanoparticle electrode, MgCo_2_O_4_ nanoflake electrode, and Ni(OH)_2_@MgCo_2_O_4_ nanosheet composites at a scan rate of 5 mV s^−1^; (**b**) comparison of the GCD curves of the electrodes at a current density of 1 A g^−1^; (**c**,**e**) CV curves of the Ni(OH)_2_@MgCo_2_O_4_ nanosheet composites and MgCo_2_O_4_ nanoflake electrodes at varied scan rates; (**d**,**f**) GCD curves of the Ni(OH)_2_@MgCo_2_O_4_ nanosheet composite and MgCo_2_O_4_ nanoflake electrodes at different current densities.

To better evaluate the electrochemical behaviors of Ni(OH)_2_ nanoparticle electrodes, MgCo_2_O_4_ nanoflake electrodes, and Ni(OH)_2_@MgCo_2_O_4_ nanosheet composites, CV, GCD, and EIS measurements were conducted using a three-electrode configuration in a 2 M KOH aqueous electrolyte solution (Figure 6). Figure 6a shows the results of the CV tests of as-developed electrodes of the Ni(OH)_2_ nanoparticle, MgCo_2_O_4_ nanoflake electrodes, and Ni(OH)_2_@MgCo_2_O_4_ nanosheet composites at a scanning rate of 5 mV s^−1^. All electrode samples possessed an obvious couple of reversible redox peaks, demonstrating that active electrodes have typical battery-type characteristics. Particularly, as depicted in the CV test results shown in Figure 6a, the specific area of the Ni(OH)_2_@MgCo_2_O_4_ nanosheet composite was much bigger than that of the Ni(OH)_2_ nanoparticle electrode and MgCo_2_O_4_ nanoflake electrode at similar scan rates, revealing the notable capacitance of Ni(OH)_2_@MgCo_2_O_4_ nanosheet composites. Figure 6b depicts the GCD plots of the Ni(OH)_2_ nanoparticle electrode, MgCo_2_O_4_ nanoflake electrode, and Ni(OH)_2_@MgCo_2_O_4_ nanosheet composites at an identical current density of 1 A g^−1^. Figure 6d,f display the GCD curves of the Ni(OH)_2_@MgCo_2_O_4_ nanosheet composite and MgCo_2_O_4_ nanoflake electrodes at different current densities. Based on the GCD tests in Figure 6d,f, the C_s_ of the Ni(OH)_2_@MgCo_2_O_4_ nanosheet composites was measured as 1287 F g^−1^ at 1 A g^−1^. At higher current density values of 2, 5, 10, and 20 A g^−1^, the Ni(OH)_2_@MgCo_2_O_4_ nanosheet composites also reached good capacitances of 1071, 926, 661, and 459 F g^−1^, respectively (Figure 6d), which were attributed to the large specific area and excellent ion interlayer exchanges of the composites.

Figure 6c,e display the CV test results for the MgCo_2_O_4_ nanoflake electrode and Ni(OH)_2_@MgCo_2_O_4_ nanosheet composites at scanning rates ranging from 5 to 25 mV s^−1^. The CV tests show that the specific area of the Ni(OH)_2_@MgCo_2_O_4_ nanosheet composites was larger, indicating the C_s_ of the Ni(OH)_2_@MgCo_2_O_4_ nanosheet composites was higher.

**Figure 7:**
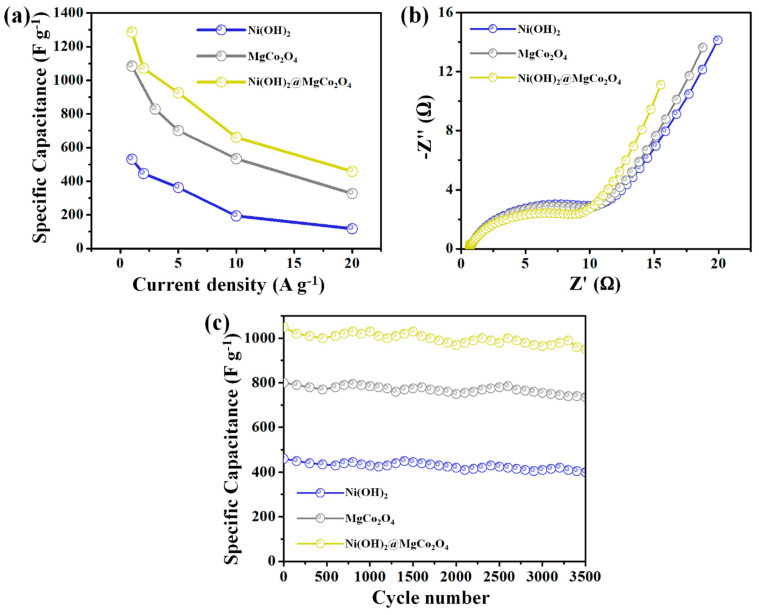
Electrochemical characterization of Ni(OH)_2_ nanoparticle electrode, MgCo_2_O_4_ nanoflake electrode, and Ni(OH)_2_@MgCo_2_O_4_ nanosheet composites: (**a**) specific capacitances of as-developed active electrodes at different current densities; (**b**) Nyquist plots of EIS; (**c**) cycling performance tests over 3500 long cycles at a current density of 2 A g^−1^.

The specific capacitances of the Ni(OH)_2_@MgCo_2_O_4_ nanosheet composites, MgCo_2_O_4_ nanoflake electrodes, and Ni(OH)_2_ nanoparticle electrodes based on the GCD tests were determined to be 1287 F g^−1^, 1084 F g^−1^, and 531 F g^−1^, respectively, at a current density of 1 A g^−1^ (Figure 7a). Importantly, the Ni(OH)_2_@MgCo_2_O_4_ nanosheet composite electrode was found to exhibit notable rate capabilities with 74.5% retention of the starting capacitances at 20 A g^−1^.

EIS measurements were conducted on the samples, and the corresponding Nyquist plots are presented in Figure 7b. The outline of the impedance spectra possesses three parts. In the high-frequency area, the real axis with intersections reveals the electrolyte’s resistances (Rs), including the internal resistance, the interface resistance, and ionic resistance of the electrolytes between the working electrodes and current collector [38]. The semicircle diameters give the charge transfer impedances (Rct) [39]. The line slope in the lower-frequency region is attributed to the Warburg resistance (Rw), which is close to OH^−^ in the aqueous solution [40,41]. We can see that the Ni(OH)_2_@MgCo_2_O_4_ nanosheet composite sample has abundant vertical lines and semicircles with a smaller diameter than those of the MgCo_2_O_4_ nanoflake electrodes and Ni(OH)_2_ nanoparticle electrodes, indicating rapid electron transportation kinetics and high diffusion ion rates. The EIS outcomes reveal that the Ni(OH)_2_@MgCo_2_O_4_ nanosheet composites had lower *R*s (0.59 Ω) and *R*ct (0.07 Ω) compared to the MgCo_2_O_4_ nanoflake electrode (*R*s was 0.81 Ω and *R*ct was 0.36 Ω) and Ni(OH)_2_ nanoparticle electrode (*R*s was 0.93 Ω and *R*ct was 0.51 Ω), indicating superior electrical conductivities and fast electron transportation kinetics.

Cycling stability is also a crucial factor in studying the characteristics of SCs. Thus, continuous charge/discharge procedures were maintained for Ni(OH)_2_@MgCo_2_O_4_ nanosheet composites, MgCo_2_O_4_ nanoflake electrodes, and Ni(OH)_2_ nanoparticle electrodes at a current density of 2 A g^−1^ over 3500 long cycles. As illustrated in Figure 7c, the Ni(OH)_2_ nanoparticles and MgCo_2_O_4_ nanoflake electrodes retained 78.3% and 81.2% of their starting capacitances, respectively, over 3500 cycles. However, the hybrid Ni(OH)_2_@MgCo_2_O_4_ nanosheet composite electrode (with an initial specific capacitance of 1071 F g^−1^) retained 85.6% of its initial capacitance after 3500 long cycles. The unique hybrid constructions achieved outstanding cycling stability results. The specific capacitance values of the Ni(OH)_2_@ MgCo_2_O_4_ electrode were compared with those found in previous studies, as shown in Table 1.

## 4. Conclusions

In summary, we successfully fabricated a Ni(OH)_2_@MgCo_2_O_4_ nanosheet composite electrode on Ni foam using a facile two-step hydrothermal route followed by annealing techniques. The as-synthesized sample electrodes achieved notable electrochemical activities due to their synergistic effects. Combining the synergistic effects of the outstandingly aligned MgCo_2_O_4_ nanoflakes and unique specific area of Ni(OH)_2_ nanoparticles, the as-developed Ni(OH)_2_@MgCo_2_O_4_ nanosheet composite demonstrated a superior C_s_ of 1287 F g^−1^ at a current density of 1 A g^−1^ and rate capabilities superior to those of MgCo_2_O_4_ nanoflakes and Ni(OH)_2_ nanoparticle electrodes alone. Moreover, the Ni(OH)_2_@MgCo_2_O_4_ nanosheet composite showed a notable cyclic stability of 85.6% over 3500 long cycles. These features of battery-type electrode materials with outstanding electrochemical performances demonstrate their promise as high-performance SCs.

## Figures and Tables

**Table 1:** Comparison of electrochemical performance of Ni(OH)_2_@ MgCo_2_O_4_ electrode with earlier reported studies on a three-electrode system.

Electrode Materials	Electrolyte	Specific Capacitance (F g^−1^)/Current Density	Cycles (Stability)	Ref.
MgCo_2_O_4_@MnO_2_	2 M KOH	852.5 F g^−1^ at (1 A g^−1^)	-	[38]
CeO_2_@MnO_2_	1 M Na_2_SO_4_	255 F g^−1^ at (1 A g^−1^)	3000 (90.1%)	[39]
MgCo_2_O_4_ nanosheets	2 M KOH	947 C g^−1^ (2 A g^−1^)	5000 (96%)	[40]
Double-urchin-like MgCo_2_O_4_	3 M KOH	508 F g^−1^ (2 A g^−1^)	2000 (95.9%)	[41]
1D MgCo_2_O_4_	-	752 F g^−1^ (2 mA cm^−2^)	-	[42]
MgCo_2_O_4_ nanocone arrays	1 M Na_2_SO_4_	750 F g^−1^ (1 A g^−1^)	1000 (84%)	[43]
Porous MgCo_2_O_4_ nanoneedle	-	804 F g^−1^ (1 A g^−1^)	2000 (87%)	[44]
MgCo_2_O_4_	2 M KOH	321 F g^−1^ (0.5 A g^−1^)	-	[45]
Urchin-like MgCo2O4@PPy	-	1076.9 F g^−1^ (1 A g^−1^)	1000 (97.4%)	[46]
Ni(OH)_2_@MgCo_2_O_4_	2 M KOH	1287 F g^−1^ at 1 A g^−1^	3500 (85.6%)	This work

## Data Availability

No new data were created or analyzed in this study. Data sharing is not applicable to this article.

## Supplementary Materials

The following supporting information can be downloaded at: https://www.mdpi.com/article/10.3390/nano13081414/s1, Figure S1: XPS full spectra of MgCo_2_O_4_ nanoflakes grown on Ni foam.
