# Supplementary material for: Hierarchically Developed Ni(OH)2@MgCo2O4 Nanosheet Composites for Boosting Supercapacitor Performance
Source: Nanomaterials (Basel). 2023 Apr 19;13(8):1414. doi: 10.3390/nano13081414 (PMC10147020; doi:10.3390/nano13081414)
Supplement: Supplementary file 1 [file nanomaterials-13-01414-s001.zip › nanomaterials-2319235-supplementary.pdf]

Supplementary Materials

Hierarchically Developed $Ni(OH)_2@MgCo_2O_4$ Nanosheet Composites for Boosting Supercapacitor Performance

Hammad Mueen Arbi [1,†], Ganesh Koyyada [2,†], Yedluri Anil Kumar [3,4], Dasha Kumar Kulurumotlakatla [5], Jae Hong Kim [2], Md Moniruzzaman [6,*], Salem Alzahmi [3,4,*] and Ihab M. Obaidat [1,4,*]

1 Department of Physics, United Arab Emirates University, Al Ain P.O. Box 15551, United Arab Emirates
2 Department of Chemical Engineering, Yeungnam University, 214-1, Daehak-ro 280, Gyeongsan 712-749, Republic of Korea
3 Department of Chemical & Petroleum Engineering, United Arab Emirates University, Al Ain P.O. Box 15551, United Arab Emirates
4 National Water and Energy Center, United Arab Emirates University, Al Ain P.O. Box 15551, United Arab Emirates
5 Graduate School of Convergence Science, Pusan Nationfivel University, San 30 Jangjeon-dong, Geumjeong-gu, Busan 609-735, Republic of Korea
6 Department of Chemical and Biological Engineering, Gachon University, 1342 Seongnam-daero, Seongnam-si 13120, Republic of Korea
* Correspondence: mani57chem@gachon.ac.kr (M.M.); s.alzahmi@uaeu.ac.ae (S.A.); iobaidat@uaeu.ac.ae (I.M.O.)
† These authors contributed equally to this work.

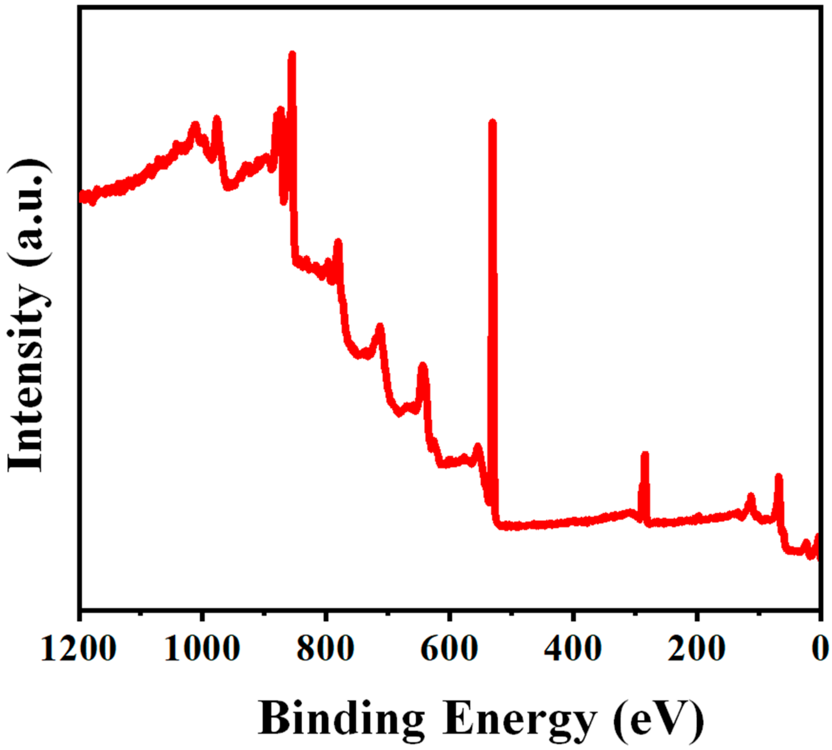

Figure S1. XPS full spectra of $MgCo_2O_4$ nanoflakes grown on Ni foam.
